# Old problem surfaces with new technology: Twiddler’s syndrome in Barostim NEO

**DOI:** 10.1186/s44348-024-00010-9

**Published:** 2024-08-02

**Authors:** Anila Rao, Lakshmi Rao, Akarsh Parekh, Surya Rao

**Affiliations:** 1 Graduate Medical Education, Michigan State University, Lansing, MI USA; 2 Department of Cardiology, McLaren Macomb Medical Center, 1000 Harrington Street, Mount Clemens, MI 48043 USA; 3 Department of Cardiology, McLaren Oakland Medical Center, Pontiac, MI USA; 4 Department of Cardiology, AdventHealth New Smyrna, New Smyrna Beach, FL USA

Barostim NEO provides baroreflex activation therapy for advanced heart failure patients by simulating the carotid barorecptors through electrical pulses from a lead attached to the carotid artery [1]. The most recent clinical trial supporting the use of Barostim NEO published in 2016, the baroreflex activation therapy for heart failure trial presented Barostim NEO as a device option beyond guideline-directed medical therapy (GDMT) for our patient’s with heart failure with reduced ejection fraction (HFrEF) with EF ≤ 35% with New York Heart Association II–III symptoms that were symptomatic despite GDMT [2]. The study demonstrated the potential reduction in hospitalization with Barostim implantation in regards to heart failure hospitalizations, length of stay and reduction in serious cardiovascular events [3]. As the implantation rates of Barostim increases given its benefit in our heart failure patient it is important to be aware of potential complications that can arise as a result. Thus, we present a rare case of delayed presentation of Twiddlerʼs syndrome in Barostim.

**Fig. 1:**
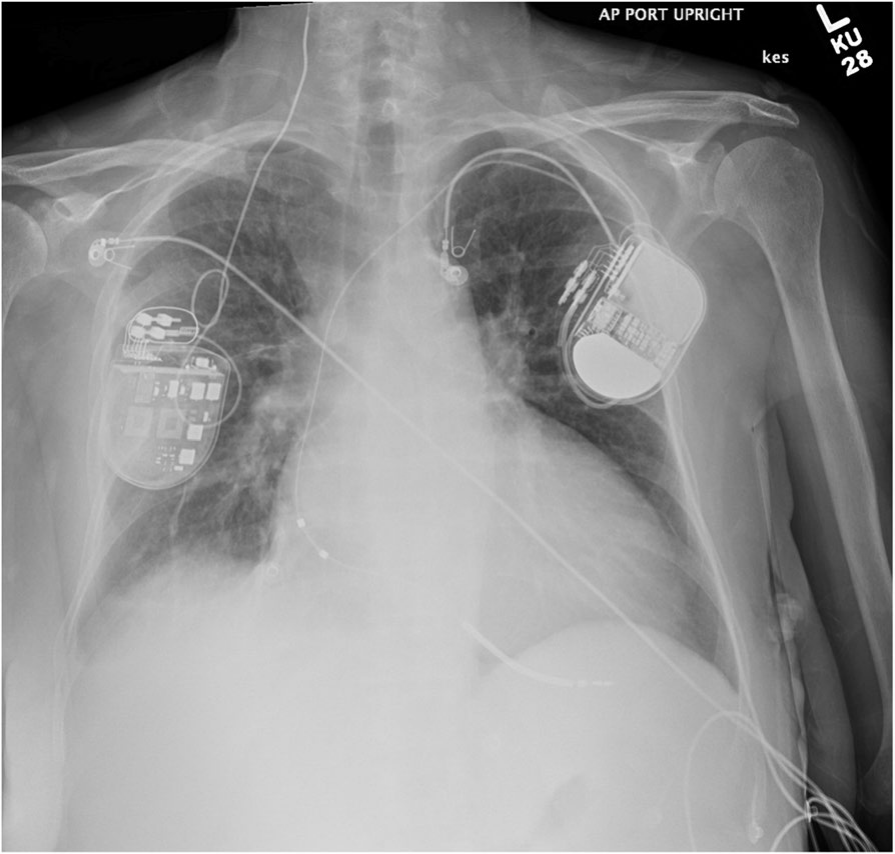
Chest X-ray. Post-implantation of Barostim NEO device with intact right carotid anchored suture wing of the non-isodiametric lead

**Fig. 2:**
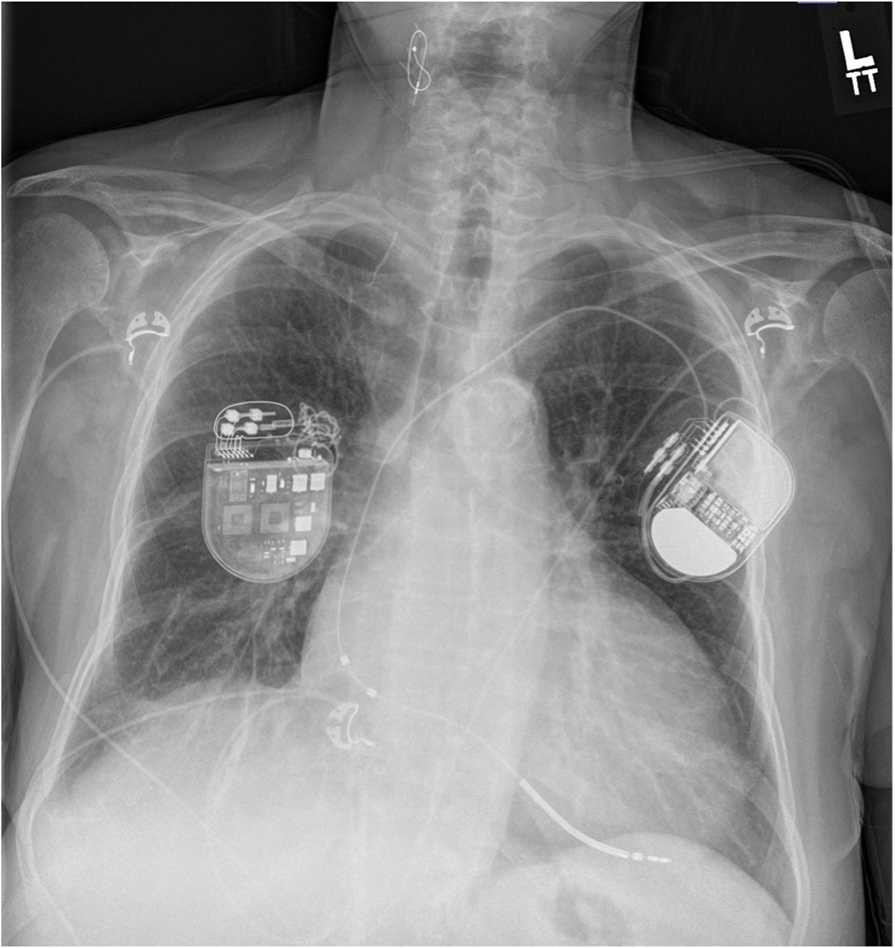
Chest X-ray. Eight months post-implantation of Barostim NEO device with fractured lead. The proximal lead demonstrates significant coiling, twisting, and retraction consistent with Twiddler’s syndrome

A 65-year-old female with a history of HFrEF status post implantable cardioverter-defibrillator on maximally tolerated guideline directed medical therapy presented with dyspnea. She previously had recurrent admissions to the hospital for HFrEF exacerbation; thus, prompting implantation of Barostim (Fig. 1). After implantation, she experienced no further heart failure admissions. 8 months post-implantation, she was admitted with acute decompensated heart failure. On chest X-ray, a fractured Barostim lead was noted (Fig. 2). Computed tomography of the neck revealed the proximal end of the lead was twisted and retracted into the pocket due to Twiddler’s syndrome. Because of the non-isodiametric design of the Barostim lead and the anchored suture wing of the Barostim device, there was little to no tension transmitted to the right carotid, and the right carotid remained intact. She was treated for her HFrEF exacerbation and is scheduled for reimplantation of the Barostim device.

Twiddlerʼs syndrome is a known complication of implantable devices that often occurs early after implantation due to generator manipulation [2]. Barostim lead design reduces the risk of carotid injury when tension is put on the lead with manipulation [1]. Despite typically occurring in newly implanted devices, this complication can occur in a delayed presentation as seen in our patient.
